# The Institutional Worker

**Published:** 1912-03-30

**Authors:** 


### THE INSTITUTIONAL WORKER

**Being a Special Supplement to "The Hospital"**

### Editor's Notices.

**Contributions:**

Contributions should be written, or preferably typed, on one side of the paper only, and all articles sent in are accepted upon the distinct understanding that they are forwarded to THE HOSPITAL only.

The Editor cannot undertake to return MSS. not used, but when a stamped directed envelope is enclosed the MSS. may be returned if a special request is made.

Accepted articles and paragraphs of news will be paid for after publication at the scale rate.

**Address:**

To prevent delay all contributions and letters on editorial business must be addressed exclusively to the Editor, "The Hospital," 29 Southampton Street, Strand, London, W.C. It is important that this regulation shall be **strictly observed.**

**Correspondence:**

Correspondence on all subjects is invited. The name and address of correspondents must be given as a guarantee of good faith, but not necessarily for publication.

**Special Articles:**

Special articles are invited, and questions, inquiries, and paragraphs upon all matters relating to the work, administration, and management of general, special, mental, fever, cottage and convalescent hospitals, sanatoria, homes, institutions, societies and organisations for the treatment or care of the sick, injured and dependants of all classes. Every matter affecting the interests and welfare of the staff of all grades working in these institutions will receive special consideration.

**Administrative Medicine, Research and Items of News:**

Special terms will be paid for approved articles on subjects in this wide field by experts who have made some section of it their special study and interest. We wish to give prominence to every movement tending to advance accuracy of diagnosis, efficiency in treatment, and the development of modern methods to eradicate disease and promote the welfare of the suffering.

**A Bureau of Information:**

We invite inquiries and applications for information and help in providing for that numerous class of sufferers who require special care or house-room which they cannot obtain in their own homes or secure for themselves. We cannot, however, prescribe, or recommend practitioners.

**Books for Review:**

Publishers are particularly requested to send advance proofs of any new books of importance whenever possible, as the Editor has made arrangements to publish immediate reviews on a new plan.

**Photographs, Plans, Blocks, and Illustrations:**

It is requested that wherever possible MSS. may be accompanied by illustrations in any of the above forms. The name of the sender and of the article to which it belongs should be written on the back of each photograph, drawing, or block for purposes of identification.

**Local Papers:**

Newspapers containing reports or news paragraphs should be marked and addressed to the Sub-Editor.

**The Coupon System:**

A coupon will be found at the bottom of the third inside page of the cover each week. This coupon must be attached to every question or inquiry to which an answer is desired in THE HOSPITAL.

### An Increase in Advertisements.

THE value of *The Institutional Worker* as a medium for announcing vacancies in every department of Institutional work is evidenced by the greatly increasing number of advertisements that are appearing from week to week in this section of THE HOSPITAL. No more adequate proof of the usefulness of *The Institutional Worker* can be desired than the fact that Authorities of some of the most important Institutions in the United Kingdom employ its columns again and again whenever they have a vacancy to fill upon their staffs. It also provides exceptional facilities for every class of announcement affecting Institutional Life and Work, and as a means of securing re-engagements in any section of the Institutional, Health, and Sanitary Services it is absolutely incomparable, since no other journal possesses such a wide and comprehensive circulation in these directions as THE HOSPITAL. If you are looking for a post an announcement of twenty-one words, costing the small sum of 1*s.*, may save you many hours of anxious thought, and will certainly bring your requirement immediately before those most likely to need your services.

This form may be used for Appointments Wanted advertisements, and when filled up should be posted to the *Manager*, THE HOSPITAL,
28 and 29 Southampton Street,
Strand, W.C.

Appointments Wanted, 21 words ... 1s. 0d.

| | | |
|---|---|---|
| | | |
| | | |
| | | |
| | | |
| | | |
| | | |
| | | |
| | | |

### Manager's Notices.

**Letters relating to the publication, sale, and advertising departments of "The Hospital" must be addressed to The Manager, "The Hospital," The Hospital Building, 28 and 29 Southampton Street, London, W.C.**

Subscriptions may begin at any time, and are payable in advance. Cheques and Post-Office orders should be crossed London County and Westminster Bank, Covent Garden branch, and made payable to the Manager of THE HOSPITAL.

RATES OF SUBSCRIPTION (payable in advance).

| | | |
|---|---|---|
| **UNITED KINGDOM.** | | |
| Three Months (including Postage) | ... ... ... ... | 2s. 0d. |
| Six Months | do. ... ... ... ... | 4s. 0d. |
| Twelve Months | do. ... ... ... ... | 6s. 6d. |
| **FOREIGN AND COLONIAL.** | | |
| Three Months (including Postage) | ... ... ... ... | 2s. 6d. |
| Six Months | do. ... ... ... ... | 5s. 0d. |
| Twelve Months | do. ... ... ... ... | 8s. 8d. |

**Advertisements:**

To ensure insertion, all advertisements for THE HOSPITAL must reach the Manager not later than Wednesday morning in each week. For scale of charges see pages vii and 2.

**Remittances:**

**It is especially requested that remittances be made by cheque or Postal Order, and in halfpenny stamps only when the amount is under sixpence.**

### OFFICIAL APPOINTMENTS VACANT.

### Poor-Law Vacancies.

Assistant Cook (£24), Bermondsey Guardians. Matron of the Workhouse, Ladywell Road, Lewisham, S.E. April 25.
Assistant for Children's Homes (£18), Taunton Union. Mr. W. F. B. Dawe, C. to G., Taunton. March 30.
Assistant Male Attendant (£30), Southampton Union. Mr. A. J. Walden, C. to G., Southampton. March 30.
Assistant Matron (£20), Stow Union. Mr. H. E. Wilkes, C. to G., Stowmarket. April 1.
Assistant Nurse (£25), Wisbech Union. Mr. T. H. Stockdale, C. to G., Wisbech. March 30.
Assistant Nurse (£20), Horsham Union. Mr. A. C. Coole, C. to G., Horsham. April 1.
Assistant Nurse (£25), Abingdon Union. Mr. B. Challenor, C. to G., Abingdon. April 2.
Assistant Nurse (£25), Ticehurst Union. Mr. J. C. L. Andrews, C. to G., Wadhurst. April 3.
Assistant Nurse (£20), Bodmin Union. Mr. J. Pethybridge, C. to G., Bodmin. April 12.
Assistant Nurse (£25), Faversham Union. G. Tassell, C. to G., Union Offices, Faversham. April 6.
Assistant Nurse (£28), Bromley Union. E. Haslehurst, C. to G., Union Offices, Park House, Bromley, Kent. April 8.
Assistant Nurse (£25), Pontypridd Union. W. Spickett, C. to G., Union Offices, Pontypridd. April 11.
Assistant Nurse (£30), Llanelly Union. D. C. Edwards, C. to G., Union Offices, Llanelly. April 8.
Assistant to Clerk (£72), Kensington and Chelsea School District. Mr. H. D. Aslett, Clerk to the Managers, 241 King Street, Hammersmith. April 4.
Assistant to Matron (£20), Freebridge Lynn Union. Mr. W. Cross, C. to G., King's Lynn. April 8.
Bathman and Barber (£28), Hunslet Union. Mr. F. W. Mee, C. to G., Hunslet. April 3.
Case Paper Clerk (£80), Christchurch Union. Mr. A. Druitt, C. to G., Christchurch. March 30.
Charge Nurse (£36), Grimsby Union. Mr. J. F. Wintringham, C. to G., Grimsby. April 1.
Charge Nurse (£30), West Ham Union. Mr. T. Smith, C. to G., Leytonstone, N.E. April 10.
Charge Nurse (£32), Bedford Union. W. Payne, C. to G., 115 High Street, Bedford. March 30.
Charge Nurse (£32), Huddersfield Union. E. A. Rigby, C. to G., Union Offices, Ramsden Street, Huddersfield. April 11.
Charge Nurse (£30), West H[illegible]m Union. T. Smith, C. to G., Union Road, Leytonstone, N.E. April 10.
Children's Attendant (£1[illegible]), Bedford Union. Mr. W. Payne, C. to G., Bedford. March 30.
Clerk (£52), Bromsgrove Union. Mr. H. D. Holloway, C. to G., Bromsgrove. April 1.
Cook and Assistant Matron (£22), Alnwick Union. Mr. H. W. Walton, C. to G., Alnwick. April 4.
Female Assistant Cook (£30), Sheffield Union. Mr. A. E. Booker, C. to G., Sheffield. April 3.
Female Assistant Relieving Officer (£75), Chesterfield Union. R. F. Hartwright, C. to G., Union Offices, Chesterfield. April 12.
Female Attendant (£25), Poplar Guardians. Mr. G. H. Lough, C. to G., 45 Upper North Street, Poplar. April 1.
Female Attendant (£22 10s.), Stourbridge Union. Mr. G. F. James, C. to G., Stourbridge. April 4.
Female Attendant (£25), Leeds Union. Mr. J. H. Ford, C. to G., Leeds. April 4.
Female Cook (£35), Leeds Union. Mr. J. H. Ford, C. to G., Leeds. April 4.
Female Industrial Trainer (£26), Southampton Union. Mr. A. J. Walden, C. to G., Southampton. March 30.
Foster-Mother (£25), Taunton Union. Mr. W. F. B. Dawe, C. to G., Taunton. March 30.
General Assistant and Laundress (£20), Shifnal Union. Mr. H. R. Phillips, C. to G., Shifnal. March 30.
Laundress (£25), Lancaster Union. Mr. W. D. Ball, C. to G., Lancaster. April 2.
Male Attendant (£30), Stoke-on-Trent Union. Mr. T. Wood, C. to G., Stoke-on-Trent. April 4.
Male Lunatic Attendant (£30), Tynemouth Union. Mr. T. Percival, C. to G., North Shields. March 30.
Master's Clerk and Storekeeper (£30), Steyning Union. Mr. A. Flowers, C. to G., Shoreham-by-Sea. April 1.
Master and Matron (£90 and £50), Bucklow Union. The C. to G., Knutsford. April 3.
Matron's General Assistant (£20), Potterspury Union. Mr. W. R. Parrott, C. to G., Stony Stratford. April 3.
Matron's Assistant (£25), Glanford Brigg Union. Mr. F. C. Hett, C. to G., Brigg. April 10.
Nurse (£33), Ticehurst Union. Mr. J. C. L. Andrews, C. to G., Wadhurst. April 3.
Nurse (£40), Plympton St. Mary Union. Mr. J. W. Bickle, C. to G., Westwell Street, Plymouth. April 11.
Nurse (£25), Worksop Union. J. S. Whall, 66 Bridge Street, Worksop. April 9.
Nurse (£25), S. Manchester Guardians. The Matron, Sanatorium, "Plas Uchaf," Abergele, N. Wales.
Nurses (£25), Lymington Union. J. D. Rawlins, C. to G., Lymington. April 3.
Nurses (£32), Leigh Union. A. Hayward, C. to G., Union Offices, Leigh, Lancs. April 2.
Porter (£25), Midhurst Union. Mr. J. M. Furneaux, C. to G., Midhurst. March 30.
Porter and Cook (married couple) (£25 and £20), Lambeth Guardians. The C. to G., 128 Brook Street, Kennington, S.E. April 1.
Porter and Cook (married couple) (£40 joint), Shepton Mallet Union. Mr. A. E. Nalder, C. to G., Shepton Mallet. April 3.
Probationer (£12), Warwick Union. Mr. C. H. Passman, C. to G., Leamington. April 2.
Relieving Officer and Collector (£150), Epsom Union. Mr. A. G. Ebbutt, C. to G., Epsom. April 2.
Sister (£32), Tynemouth Union. T. Percival, C. to G., Guardians Hall, North Shields. March 30.
Superintendent and Matron of Cottage Homes (£50 and £35), Elham Union. Mr. E. Lovick, C. to G., 29 Bouverie Square, Folkestone. April 2.
Superintendent Nurse (£45), Stockton Union. Mr. F. B. Watson, C. to G., Stockton-on-Tees. April 3.
Superintendent Nurse (£60), Coventry Union. Mr. E. A. Evans, C. to [illegible], Coventry. April 13.

### Institutional and Administrative Vacancies.

Housekeeper (£50), Prince of Wales's General Hospital, Tottenham. The Chairman of the House Committee.
Matron (£60), Royal Victoria Hospital, Bournemouth. Gordon Martyr, Secretary. April 8.
Sister (£28), Northampton General Hospital. Matron.

### Municipal Vacancies.

Accountant (£150), Anglesey C.C. Mr. J. R. Roberts, Clerk to the Council, Llangefni. April 2.
Assistant Land Agent (£120), Dorset C.C. Mr. E. A. Ffooks, Clerk to the Council, Dorchester. April 10.
Assistant Nurse (£25), Blean R.D.C. Isolation Hospital. J. P. Burch, Clerk, 39 Castle Street, Canterbury. April 2.
Assistant to Water Engineer (£110), Warrington T.C. Mr. J. L. Whittle, Town Clerk, Warrington. March 30.
Deputy Town Clerk (£1,000), Manchester T.C. Mr. T. Hudson, Town Clerk, Manchester. March 30.
Fourth Sanitary Inspector (£100), Edmonton U.D.C. Mr. W. F. Payne, Clerk to the Council, Edmonton. April 1.
Health Visitor (£80), County Borough of Huddersfield. J. H. Field, Town Clerk, Huddersfield. March 30

